# Opportunistic cognitive screening in Sweden: What the tests mean and do for patients and healthcare professionals

**DOI:** 10.1177/14713012211035373

**Published:** 2021-08-11

**Authors:** Kristin Zeiler, Göran Karlsson, Martin Gunnarson

**Affiliations:** Department of Thematic Studies: Technology and Social Change, and the Centre for Medical Humanities and Bioethics, 272059 Linköping University, Linköping, Sweden; Motala Hospital, 4564 Region Östergötland, Sweden; Department of Thematic Studies, Technology and Social Change, Centre for Medical Humanities and Bioethics, 272059 Linköping University, Linköping; The Centre for Studies in Practical Knowledge, Södertörn University, Huddinge, Sweden

**Keywords:** Cognitive impairment, opportunistic screening, subjectivity, lived experience, Sweden, healthcare professionals’ and patients’ perspectives

## Abstract

Since 2017, opportunistic screening for cognitive impairment takes place at the geriatric ward of a local hospital in Sweden. Persons above the age of 65 who are admitted to the ward, who have not been tested for cognitive impairment during the last six months nor have a previously known cognitive impairment, are offered the Mini-Mental State Examination and the Clock-Drawing Test. This article analyses what the opportunistic screening practice means for patients and healthcare professionals. It combines a phenomenologically-oriented focus on subjectivity and sense-making with a focus that is inspired by science and technology studies on what the tests become within the specific context in which they are used, which allows a dual focus on subjectivity and performativity. The article shows how the tests become several different, not infrequently seemingly contradictory, things: an offer, an important tool for knowledge-production, something unproblematic yet also emotionally troubling, something one can fail and an indicator that one belongs to a risk group and needs to be tested. Further, the article shows how the practice is shaped by the sociocultural context. It examines the role of the affective responses to the test for subjectivity – particularly patient subjectivity – and offers a set of recommendations, if this practice were to expand to other hospitals.

## Introduction

Opportunistic screening for cognitive impairment involves offering tests to a population that seeks health care for other reasons than a concern with their cognition. This article analyses what the opportunistic screening practice means for patients and healthcare professionals, when being offered at a geriatric ward at a local hospital in Sweden. We are interested in what this practice can help to achieve – that is, its performative effects – and how it can affectively help shape subjectivity. The article combines a phenomenologically-oriented focus on subjectivity and sense-making with a focus that is inspired by science and technology studies (hereafter referred to as STS) on what the tests become within the specific context in which they are used, as narrated by the occupational therapists, doctors and patients we have interviewed. These perspectives have rarely been combined (see, however, Zeiler, 2020; Hoel and Carrusi, 2015), and doing so allows for a combined focus on performativity and subjectivity.

### Opportunistic screening for cognitive impairment: The case

Opportunistic screening is not generally offered at geriatric wards in Sweden, but it was introduced at the geriatric ward of a local hospital in Sweden in 2017. Persons above the age of 65 who are admitted to this ward, who have not been tested for cognitive impairment during the last six months nor have a previously known cognitive impairment, are offered the Mini-Mental State Examination (MMSE) and the Clock-Drawing Test (CDT).^1^ At the ward, occupational therapists inform patients who meet these criteria about the tests, and ask if they want to undergo the tests. Taking part in opportunistic screening is voluntary. The percentage of patients who have taken the tests, out of all patients admitted to the ward, have varied between ca 10–26% since 2017, when the screening practice was introduced (see Table 1). Occupational therapists perform the tests, assess them and talk to the patients about their results. Between 2019 and 2020, five occupational therapists performed the majority of the tests.

**Table 1.** Number of patients in total, tested and with a test result ≤24 on MMSE/who failed the CDT.

Year	Number of patients admitted to the ward (inpatient care)	Number of patients tested for cognitive impairment	Number of patients with a test result ≤24 on MMSE or who failed the CDT
2017	667	64	20
2018	768	197	78
2019	800	160	48
2020	734	112	43

Note: CDT: Clock-Drawing Test.

Before patients are discharged from the hospital, they also discuss the tests and their results with a doctor. During this consultation, the doctor records the results of the tests in the patient’s medical notes. If a patient has a test result of 24 or below on the MMSE or does not pass the CDT, this is specifically notified in the notes, so that primary care professionals can note these test results and consider whether any follow-up testing may be offered. Scoring low on these tests may, but need not, indicate that further cognitive assessment as part of a basic dementia examination is called for. The percentage of patients with a test result of 24 or below on the MMSE or who have not passed the CDT, out of all tested, has varied between 30 and 40% (see Table 1).

One of the objectives of the opportunistic screening practice at this hospital in Sweden is to identify patients who may benefit from such further examinations. The practice has also been motivated by the view that cognitive impairment is an often underdiagnosed condition that is associated with increased mortality among people above the age of 60 (Torisson et al., 2012).

The screening practice at the hospital in Sweden is similar to a practice in Norway, where tests such as the MMSE and CDT have been offered to persons over the age of 65 when they are admitted to geriatric wards for other reasons than concerns with their cognition (Krohne et al., 2011: 680). It is also similar to a practice in England in which persons over the age of 75 who are admitted to hospital, unplanned and for more than 72 h, are asked if they have been forgetful during the past 12 months to the extent that it has significantly affected them in their daily life. Those who answer this question affirmatively are offered the Abbreviated Mental Test (Department of Health, 2012). This practice, Barret and Burns have explained, ‘may be considered a type of screening’, and it has been justified by its target of a ‘high-risk population’ of persons above the age of 75 (Barret and Burns, 2014: 9). One Norwegian (Krohne et al., 2011) and one British (Burn et al., 2019) study have explored these practices from the perspective of tested subjects. These studies show that those who are screened have a wide range of experiences of this, from relief to frustration and feelings of failure and shame (Burn et al., 2019; Krohne et al., 2011). Our study confirms the range of affective experiences found in the two previous studies, and adds the combined STS-phenomenology inspired perspective, which allows for a deeper analysis of what the test becomes, how the practice is shaped by the sociocultural context and the role of affect for subjectivity, when being tested.

## Methods and research ethics

The empirical data that form the basis of our analysis consists of nine interviews with patients and seven interviews with healthcare professionals (five occupational therapists and two doctors). Inclusion criteria for patients to be interviewed were that they had taken both tests and received an MMSE test result above 20. A test result of 21 and above was understood, at the hospital, as indicating that the patient in question could understand what the project was about and consent to an interview. Inclusion criteria for healthcare professionals to be interviewed were that they had either performed the tests or talked to patients about their tests as part of the discharge procedure from the hospital.

Interviews with patients were conducted, in their homes, when they had been discharged from the hospital, that is, after the tests (that were performed at the hospital, before patients were discharged). All interviewees were informed about the project orally and in text, and informed that they could interrupt the interview at any time without giving a reason. Patients were first informed about the project by an occupational therapist and, at a later stage, by a researcher in the research team – if the patient had said that s/he wanted more information about the project. As part of the qualitative design of the project, the goal was to do circa 8–10 patient interviews, provided that this meant that saturation was reached, that is, that no new themes emerged from the last interviews. All patients who fulfilled the inclusion criteria, from November 2019 to February 2020, were informed about the project, and asked whether they wanted to take part in an interview. 70% of the patients who were invited to take part in an interview accepted the invitation. 30% declined this invitation. The goal of 8–10 patients was reached in February 2020 (i.e. 9 patients had been interviewed). Saturation was reached. Written informed consent was obtained before every interview.

The interviews were qualitative in nature and had a semi-structured design, which meant that although they were based on interview guides with certain foci (one for the interviews with professionals and one for the interviews with patients) and open-ended questions, interviewees were also allowed to expand on the issues that they wanted to talk about or bring up issues they considered important. The interview guide for the healthcare professionals focused on their narrations of how the practice was performed; how it had changed over time; their views and experiences of offering and giving information about the tests; their views and experiences of positive and potentially more challenging aspects of offering, performing and assessing the tests; and their views on how the screening may be used in the future. These interviews were carried out by the first author. The interview guide for the patients focused on their narrations and experiences of being offered and taking the test; the thoughts and feelings that undergoing the test had aroused in them; their views and opinions about this form of testing; and what the test meant for them. These interviews were carried out by the last author. The study followed the research ethical principles of the World Medical Association Declaration of Helsinki (2018) and Swedish research ethical regulations (etikprövningsnämnd, the Swedish Research Ethics Board: approved, Ref. No. 2019-03034).^2^

The interviews were qualitative in nature and had a semi-structured design that allowed interviewees to expand on issues that they wanted to talk about. All interviews were audio recorded, transcribed verbatim and pseudonymized. All names used here are fictitious (see Table 2). A thematic analysis was then performed: the data were coded, codes were put together into broader units of analysis such as sub-themes and sub-themes were put together into themes (Braun and Clark, 2006), based on the aims of the article. The thematic analysis was combined with a focus that was inspired by discourse analysis on how the tests were talked about and how subjects and tests were discursively positioned. This approach acknowledges ageing, cognition and dementia as socioculturally situated concepts that help to shape how people understand themselves (see Willig, 2000). The first and the last authors performed separate initial codings, which were later compared in order to ensure the quality of the analysis. They then examined the data together, and finally developed the three themes presented in this article. The middle author contributed medical expertise and knowledge about the screening practice.

**Table 2.** Reasons why the interviewed patients had been admitted to the hospital.

Fictitious name	Reasons for being admitted to the hospital
Ulla	Fall accident
Ove	Fall accident
Ritva	Fall accident
Rut	Fall accident
Ingrid	Epileptic seizure followed by fall accident
Lars	Erysipelas
Klas	Erysipelas
Stig	Foot infection
Gunnar	Respiratory distress

### Theoretical perspectives

The article takes inspiration from an STS perspective, in which healthcare is understood as a knowledge-production practice that helps enact subjects, objects and specific realities in distinct ways (cf. Law, 2009; Moser, 2011). This perspective understands healthcare practices as performative, and in this way allows an analysis of what the tests become and what they can help do for the people involved in the practice. As an example, an MMSE test can be a test to assess cognitive function in a screening practice, and it can be one tool among others within a diagnostic process for dementia. The test, then, becomes different things in different healthcare practices, and the way in which these practices are performed can help to shape the distinct ways in which the test is offered, performed or declined. The way in which the tests are performed may also help to shape different conditions for what it is possible to do, be, or feel in relation to them (compare Moser, 2011).

We have combined the performative focus described above with a phenomenological eye for subjectivity, which adds the perspective of the people involved, and how these actors not only emerge as particular subjects from taking part in the testing practice but also shape and give meaning to it, and how being tested can help shape their sense of self.^3^ In our phenomenological perspective, such meaning-making takes place in and through a deep intertwinement between the self and the world, in which the self is understood as a sensing, embodied being that is capable of reflecting about herself and her surroundings, and the world is understood as a material, historical and cultural setting that the self inhabits (cf. Carel, 2008; Kontos, 2005; 2012). According to this view, the self and the world are mutually dependent: one cannot be understood without the other. Moreover, for phenomenologists, the relationship between the self and the world is not emotionally neutral but affectively charged (see, for example, Fuchs, 2015). Affectivity is here understood to be a dimension through which the world and things in it matter to us: affectivity and perception ‘comprise our sensibility, our means or manner of opening on to a world we are already “in” or “of”’ (Cataldi, 2008: 163; compare Merleau-Ponty, [1945] 2006). In this understanding, a practice such as the one studied here cannot be fully understood without taking into account the affectively charged experiences and the meaning-making carried out by the actors involved, which is shaped by and shapes their inhabitance of a world that consists of things, practices, other people, shared cultural conceptions and so on.

The combination of STS and phenomenology permits a focus on performativity and subjectivity. It enables an analysis of what the tests become within the practice as situated within a wider cultural context, and what this means for subjectivity. Since the empirical basis of this article consists of interviews, our analytical focus is on both the practice that is narrated through the interviews and how this practice is enacted – that is, shaped by the actors involved – in the interviews. We are interested not only in how opportunistic screening is carried out in practice but also in narratives about it, and we view these dimensions as intimately intertwined.

### Dementia and the broader Swedish context

Dementia is a syndrome in which memory, thinking, behaviour and the ability to perform everyday activities deteriorate. Age is its strongest known risk factor, though dementia ‘is not a normal part of ageing’ (WHO, 2019). According to the Swedish National Board of Health and Welfare, 20,000–25,000 persons per year develop dementia in Sweden, 130,000–150,000 persons live with dementia in the country, and this number is expected to double by 2050 (Socialstyrelsen, 2019). In 2019, 20% of the population in Sweden was older than 65 years (Regionfakta, 2019). As many other countries do, Sweden encourages the early diagnosis of dementia (Socialstyrelsen, 2017), but does not recommend population-based systematic screening for it. Opportunistic screening for dementia has not been assessed by the National Screening Board.

Previous studies have shown that an understanding of dementia as a one-directional loss of one’s identity or selfhood dominates the cultural consciousness of the Western world (see, e.g., Van Gorp and Vergruysse, 2012; Clarke, 2006). However, recent studies in the medical humanities in Sweden and elsewhere have offered alternatives to the conceptualisation of dementia as a ‘loss of self’ and explored remaining capabilities when living with dementia (e.g. Hydén and Antelius, 2017; Örulv, 2010). Some studies have recognised that dementia can ‘bring to the fore deeply rooted existential fears’, while at the same time stimulating the formation of self-help groups, where people with dementia and their kin support each other in such matters as managing the fear of loss of control, and the trivialisation of, and shame associated with dementia (Örulv, 2012: 10, 30). Such a multifaceted affective understanding of living with dementia also provides a background to this study, even if the practice we have studied is screening for cognitive function, generally.

## Results

The analyses resulted in three broad themes that all contain several aspects of the testing practice. The themes are: ‘What the tests become in and through the practice’, ‘The tests and the context’ and ‘What the tests and their results do and mean’.

### What the tests become in and through the practice

The first theme that we developed centred on what the tests become in the interviewees’ narrations about the practice. It was clear that the question of what the tests are is not settled once and for all, but is constantly negotiated in and through the practice itself, by the actors. The healthcare professionals explained that they never mentioned the word dementia in their conversations with patients about the tests. Instead, they stressed that the tests were not diagnostic but could merely indicate cognitive decline. In order to get this point across, they added, they used a number of alternative terms for designating the test, such as ‘paper-and-pen test’, ‘memory test’ or ‘a test for thinking ability’. Another strategy that several of the healthcare professionals used was to emphasise that the tests were ‘routine memory tests’ that were offered to ‘everyone’ on the ward. The main purpose of these strategies was to defuse and normalise the tests. One doctor explained that referring to the routine nature of the practice can, for example, prevent a patient from feeling identified as different or as having problems. Another normalising strategy was to compare and equate cognitive testing with somatic testing. One occupational therapist, Nora, used the following formulation for the practice: ‘You test for so many other things, so it’s really good to test for cognitive functions, right?’

Patients who described the tests as useful check-ups, just as one might have other check-ups, used similar formulations. Patients referred to the tests as a ‘questionnaire’ or ‘conversation’, and some patients, such Gunnar and Ove, added that it was offered to ‘persons who had reached a mature age’ to check their ‘memory functions’. Further, some patients explicitly associated the tests with dementia, even before being tested and despite the efforts taken by healthcare professionals to clarify that the tests were not tests for dementia. Lars, for example, explained that he ‘got the impression that it was sort of a basic course in senile dementia. And I thought that was positive, that one could have that, test that’.

In their reflections during the interviews, the healthcare professionals alternated between describing the tests as an advantageous offer and as something that the patients are subjected to. A related duality was that between the testing as a rather unproblematic, routine practice and as a potentially challenging one that might evoke feelings and arouse taboos relating to memory, cognitive impairment and dementia.

The potentially challenging dimension of the tests became clear in some of the occupational therapists’ narrations of how some patients responded to the offer of the tests. While some patients were described responding positively to the offer, stating ‘Oh, that’s nice, I have never been given … any check-up’, other patients were described as more hesitant and asked follow-up questions such as ‘Why would I do that [take the tests]?’, ‘Do you think I need that?’ and ‘Do you think I’m stupid?’. Occupational therapist Klara reflected on this, saying that it seems as if a few patients believe that the healthcare professionals want to ‘catch the patients out’. If this was the response from a patient, she added, she would explain that the tests were part of the ward’s routines.

The dichotomy in which the tests are unproblematic yet potentially sensitive was present also in the patient interviews. On the one hand, patients typically described the tests as a minor part of their experience of being ill and admitted to hospital. Many stated that it was ‘natural’ and ‘a good idea’ to be offered and take the test at the geriatric ward. Almost all interviewed patients described it as ‘obvious’ that they would take the test. Ulla, for example, explained that she is ‘pretty interested in things, if something happens’, which made her say ‘yes, of course I agree to be part of this’. She added that the occupational therapist ‘was so nice, so this was nothing’, and that the test ‘was about me, so I guess that was why I said yes’. Some patients, such as Ove and Lars, described the tests as particularly useful for those who might ‘brood’ over their memory, yet who would not themselves take the initiative to be tested. Further, Lars had also ‘noticed that with some things, I might not really be on the ball’, an observation that promoted him to take the test without hesitation. On the other hand, several patients also described how other patients, who fear that they might have cognitive deficiencies, may be more negative to the tests. Again this was expressed by Lars when he explained that some people ‘with minor dementia’ might have ‘a lot of suspicions and … fear that people will get at them and, well, punish them in some ways for not managing the test or, yes, … you know, and then they experience this as very negative’.

Throughout the different narrations described and cited above, the tests become different things, not infrequently seemingly contradictory things. They are enacted as both similar to and different from other tests, as both something offered and as something patients are subjected to, as – on the one hand – routine and beneficial, but – on the other hand – potentially emotionally challenging, and as being both for everyone and particularly for patients in the ‘risk zone’. These dualities lead to the tests emerging as ambiguous. This is especially so for the patients who often experience them as simultaneously valuable and worrisome. For example, when the tests are associated with dementia (which Lars suggested that they are), and dementia is understood as a fearsome one-directional loss of self (Van Gord and Vergruysse, 2011), then the value of recognising it is mixed with the worry of being diagnosed with it.

Such feelings illustrate the affective dimensions of the practice. Although taking the tests is often experienced as a minor event, it also frequently spurs affective responses, which range from worry about what had happened if one had scored low to uncertainty about whether something is actually wrong. This puts the tested persons’ subjectivity at stake; the tests are able to ‘catch the patients out’ and put them into certain wanted or unwanted categories and, at times, it seems, inform their self-understanding. The healthcare professionals are clearly aware of this, and try to manage these affective and subjectifying responses by attempting to control what the tests become, in the practice, through the defusing and normalizing strategies. However, the ambiguities are difficult to remove altogether, which will become more evident in the next section, where we take a closer look at the contextual dimensions of the practice.

### The tests and the context

The second theme that we developed centred on the ways in which the interviewees’ perceptions and experiences of cognitive testing were affected by the sociocultural context in which it took place. Here, we considered several levels of context, from the hospital setting in which the tests were performed to the perceptions of ageing, cognitive difficulties and dementia that are held by society at large.

The interviewed healthcare professionals explained that they had initially been doubtful about the idea of testing on the ward since, according to mainstream medical opinion, it should not be performed on hospitalised patients, who might suffer from temporary delirium (Burn et al., 2018; Jackson et al., 2013). Their concerns had, however, begun to dissolve when they heard of research that contradicted the mainstream opinion, saying that this form of testing can reduce mortality among patients who undergo it (Torisson et al., 2012). The scepticism became even less when the professionals had integrated the testing into their everyday work.

Patients were also concerned about the tests being performed at a hospital, after people had been admitted for other reasons, and they reflected on whether pain killers or other drugs might affect the test results. Ritva wondered whether it was ‘adequate to test this in a hospital environment with patients with somatic diseases’. ‘It might be difficult to get the correct results’, she said. Stig stated that he was tired and in pain when taking the tests and did not really see this as the ‘optimal’ situation for testing. Here, the tests are enacted in line with the medical mainstream opinion, as something that might not belong and function correctly in a hospital environment.

The context of the tests also includes the routines at the ward such as the tests being offered when the conditions were calm and quiet. Some patients were also not offered the tests, such as patients who had, for example, visual or hearing impairments or were in the later stages of cancer. The occupational therapists explained that this was based on a holistic understanding of what would be beneficial for them. Further, even though the routines state that ‘everyone’ who fulfils the criteria should be offered the test, occupational therapists described careful deliberations about to whom to offer the tests and when to offer them. These deliberations were often collegial in nature. However, while these deliberations can be understood as part of a professional judgement, the decision about who should be given the tests was a source of concern for some patients. Some patients, for example, such as Klas and Ritva, wondered if the occupational therapists ‘were checking up on them’, and chose to approach them because they judged them to be ‘good’ or ‘appropriate’ candidates for the tests. This made them doubt whether the tests were offered to everyone. Maybe, Lars wondered, stating that everyone is offered the test is ‘a really great way of saying that everyone gets to do it, but maybe they only do the tests on those who, who they suspect have something…’. This had made him wonder: whether ‘I really am getting old and that’s why they did these tests?’ Thus, the description of the tests as ‘routine’, in combination with a careful deliberation about who should be offered the tests, might have unintended effects: discovering that not everyone is asked about the tests spurred the question ‘Why me?’ among some patients.

Again, we see how the hospital setting affects what the tests become. For the group of hospitalised patients, it is possible for occupational therapists to make careful assessments about who are suitable candidates. This approach, however, reinforces the ambiguity of the tests, and they emerge, for some patients, as a worrying mix of tests offered to all and tests offered to particular, suitable candidates.

The widespread notion that cognitive deterioration and memory problems, in particular, are connected to old age also affected the patients’ perceptions and experiences of the tests. Several patients described memory problems as a natural part of getting old. Some also made the connection between old age and dementia. Lars, for example, said that he associates ‘senile dementia’ with ‘just simply getting old’. In these accounts, the wider sociocultural context is at work, in which particular connections between memory problems, dementia and old age are made. The way these connections are made here can be understood as a way of moving these phenomena out of the medical context in which the tests occur, thereby de-medicalising and normalising them.

However, the interviews made it clear that this strategy was not completely successful. On an individual level, worry about memory problems and dementia was still evident, and these concerns were in some cases enhanced by the experience of undergoing the tests. Memory problems, Lars stated, are something ‘one frets over’, and Stig described dementia as ‘just terrible’, resulting in people no longer ‘recognising their loved ones’. Taking the tests reminded Ove of his neighbour who had recently passed away with dementia, a fate he himself ‘was terrified of’.

For Sture, the testing was not disturbing until he told some friends about it. The friends associated the tests with Alzheimer’s disease, implying that that was what Sture had been tested for. This made Sture concerned, and he said, ‘I started to wonder, perhaps, a bit about that. Because I hadn’t thought about that. I thought “No”, I thought, “is it like that?” “Because they didn’t say that.” No’. Sture expressed this uncertainty and concern several times during the interview, stating at the end that ‘it might be revealed that I have it [Alzheimer’s disease]’.

Again, these accounts must be understood against the backdrop of a sociocultural context in which dementia is commonly perceived of and talked about as a gradual loss of self. In our interviews with healthcare professionals, it was clear that these contextual circumstances shaped what the tests became on the ward. Rather than a neutral check-up, the tests were associated with affectively and negatively charged phenomena such as ageing and dementia. This made talking about cognitive impairment in a ‘natural way’ ‘pretty challenging’, as occupational therapist Vera put it. Not infrequently, patients associated the mention of a memory test or memory problems with being given the diagnosis of dementia, as, for example, was the case for Ulla. Doctor Karin, for instance, compared the present understanding of being diagnosed with dementia with previous perceptions of cancer: ‘If you said “cancer”, well, then you were dead. And that’s no longer the case, and I think that it’s similar, people talk about senile dementia and that has such bad connotations, and there is […] a spectrum of dementia diseases, which all express themselves differently’. Karin added:

People talk about senile dementia or say ‘I think he has dementia’. It becomes a term of abuse, so that … Well, I think that it becomes one, and then you don’t want to get that diagnosis and this is what makes people so concerned, as I said before. ‘Is it dementia, doctor?’.

Thus, the widely shared, sociocultural understanding of dementia as a one-directional loss of self emerged in the testing practice, and was something the healthcare professionals must deal with. This involved a complicated balancing act between giving sufficient information about the tests and not frightening the patients by mentioning culturally charged terms such as ‘dementia’. However, even if such a balancing act was successful, culturally shared notions could still enter the test situation, giving rise to affectively charged experiences that influenced patients’ self-perception and their way of engaging with the world. For Lars, undergoing the tests was yet another reminder that he was not as young as he felt, while it made Ove reflectively aware of the risk of falling ill with dementia, of which he was terrified.

Here, the healthcare professionals hoped, the hospital environment might provide a setting in which such conceptions could be somewhat neutralised. It might be easier, the occupational therapist Nora said, to talk about things such as ageing, memory problems and dementia with healthcare professionals than with one’s relatives, since the former are able to conceptualise cognitive problems in similar terms as other medical problems.

### What the tests and their results do and mean

The third theme that we developed revolved around what the tests and their results can do and mean for the patients, from both patients’ and healthcare professionals’ perspectives.^4^ This involves patients’ interpretations and experiences of the tests, which may affectively shape their subjectivity, and healthcare professionals’ ways of meeting these interpretations and experiences.

Some healthcare professionals described the test results as something that could reveal something concealed. They could bring to light patients’ cognitive difficulties, and once such difficulties had been identified, patients could be given better support in their everyday life. A test result of 24 or below, in particular, was understood as a possible early signal to the primary care system that a certain patient might benefit from being offered further testing after discharge from hospital.

Some patients’ narrations were consistent with this view of the tests as offering a way of obtaining helpful information, as when patients said that the tests could function as a reminder of the need to be on the lookout for changes in one’s ability to remember. Klas, for example, described the tests as capable of raising one’s self-awareness ‘if one notices, based on the test results, that things don’t really add up anymore, one can become aware of one’s own … way of being’. This is an example of the potential effects of the tests on subjectivity; when undergoing them one can become someone who observes oneself, noting small shifts in one's abilities and reflecting on their possible meanings.

Other patients, however, questioned whether the tests could tell them anything new about their cognition. Ritva stated, laughingly, that ‘If you wants to know which stage you’re in … well, no, that’s not needed. I don’t think so. I’ll do a difficult crossword puzzle [to sort that out]. It’ll be fine’. For Ritva, being able to solve a difficult crossword puzzle provided all the information she needed about her cognitive state. Stig said that the tests had not been a problem for him, and that it would have been ‘worrying’ and ‘distressing had they [the tests] been so [a problem]’. During the interview, Stig recalled having received an appointment with the primary care system for a further cognitive check-up after his hospital stay. But, he did not think he needed it. He said that he did not mean to say that the test results were ‘uninteresting’, but that he felt that he had no cognitive difficulties, adding that ‘it was perhaps enough that I know this, myself’. These two narrations are examples of instances in which the interviewed patients used their subjectivity, their experience of who they are and what they can do, to downplay the idea that the tests provide valuable information, at least for them.

Finally, healthcare professionals emphasised the importance of telling patients about the test results in an honest and careful way. If the results indicated ‘a substantial risk of impairment’, this should be communicated, Doctor Karin stated. A low test result, Karin added, could show that someone has ‘a deficiency’ of a sort that made it difficult to perform a certain part of a test, and such a test result may indicate the need for further cognitive assessment while not implying that the person had cognitive impairment or dementia. Others emphasised the value of helping patients to realise that also a low test result can be helpful. Occupational therapist Liv explained that she was careful to take time to explain the test results, particularly to patients who had been saddened by a result of 24 or below, saying that obtaining such a result can be seen as a ‘strength’. As she put it: ‘I can explain that I won’t give a dementia diagnosis, at all, and that you can regard the results as “strengthening”, in the sense that you can get help in your everyday life’. Some patients, Liv continued, may have been concerned about their forgetfulness, wondering whether they turned the oven off or not. Such patients can receive technical aids, such as a timer that turns the oven off. When she explained this to patients, Liv said, most of them respond ‘Oh yes’. This way, she added, scoring low on the tests ‘doesn’t need to become such a big thing’. Even so, other occupational therapists such as Emelie recalled how patients had asked ‘Will I be put into an institution?’ or ‘Does this show I’m getting slow?’ when they were given their test results. Emelie emphasised that it was important to talk about such reactions with patients also, so that they can ‘let go’ of these emotions ‘a bit’ and not ‘become obsessed about this and become really worried about what the tests have said about me’.

Undergoing the tests and receiving the results may be experienced and interpreted in several ways, and this variety provided a challenge for the healthcare professionals. In the opportunistic screening practice in which they were involved, it was not ethically defensible for them to inform patients about their test results and then just let them go. As the narrations above suggest, it is necessary to meet and manage the patients’ reactions, which can relate to and alter deeply held perceptions of their selves. Therefore, as we have seen earlier, healthcare professionals tread carefully, and give time and support to patients and remain constantly aware of the contextual dimensions. Through this work, they are and become thoroughly entangled in the self-other-test-world relationship created by the practice, and their way of responding to a particular patient’s reactions can help to shape the sense of self that this patient has, as someone who has taken the test and may or may not have cognitive difficulties.

### Discussion: Insights from the study relevant to healthcare practice

Healthcare professionals’ narrations of the tests as routine tests that offer important knowledge can be read as harmonising with those of the patients’ who make sense of them as health check-ups. When this is the case, the screening practice is understood as an unproblematic and valued tool used to assess cognitive health, where cognitive health is something to check just as one may check one’s blood pressure or CRP. However, the narrations of the professionals in which patients ask whether they are stupid, and the accounts from patients of their fear of low test results, suggest that the tests can have more existentially challenging features, and the practice can feed into the way in which patients make sense of themselves as needing a ‘crash course in senile dementia’. This may take place in a context in which dementia is perceived as something to dread. Narrations that down-play the test results can also be understood against this sociocultural understanding. Down-playing the test results can then be understood to be a discursive way to create distance between oneself and one’s test results – not allowing the tests to elicit concern about one’s own cognitive health. Such down-playing, however, may also be interpreted as a way to resist the measuring mode that the tests enact, and claim agency, in the sense of the ability to make one’s own subjective assessments.

The performative perspective of this article sheds light on what the testing practice helps to do, and what the tests become, discursively, in narratives of the practice. The tests are something that is offered, an important tool used to produce knowledge and something unproblematic that may yet be emotionally troubling. They become something one can fail, and an indicator that one is at risk of getting old and needs to be tested. The phenomenological focus shifts attention to subjective meaning-making and to the self-other-test relationship created through the practice. From this perspective, the test, understood as an important tool for knowledge-production, can feed into the patients’ sense of themselves as persons who can allow such knowledge to be produced. Likewise, the test as an assessment that one may fail can help to shape the patients’ sense of self as someone who needs to be assessed. In this case, the healthcare professionals are seen as assessors. The self-other-test relationship is also affectively charged, as shown in both positive and critical narrations from the patients: the tests, while presented in a careful fashion, are not neutral for patients: they enter their lives in ways that signal that they are at risk of, or even show signs of, potentially life-changing cognitive conditions. Combining these analytic foci has allowed us to explore what the tests help to do and mean, for singular selves, on the ward, and in the cultural context in which one lives.

The opportunistic screening practice at the geriatric ward is, to our knowledge, the first of its kind in Sweden (see, however, Torisson et al., 2012). Three insights from this study will be valuable if other geriatric wards set up similar practices. First, there is a tension between giving clear and adequate information about the tests, and this information making patients concerned and worried about their cognition. Careful follow-up of the patients’ experiences of the testing, possibly a few weeks after they have been discharged, could be helpful in this respect. Second, while the MMSE contains instructions for how it should be performed, offering it at a geriatric ward when patients have been admitted for other reasons, seems to require careful deliberation on the part of the professionals. If time and resource can be set aside for such deliberations, the ability of the healthcare professionals to meet patients’ experiences of, and interpretations of, the test can be improved. Third, and relatedly, patients do notice that not everyone is offered the tests. In order to avoid concerns on the part of patients who wonder why some are offered the tests and not others, a routine for exclusion criteria could be developed.

## Notes

1 The MMSE includes tests of orientation, attention, language and memory, while the CDT tests cognitive domains such as language, executive function and visuospatial abilities.

2 The patient sub-project has been approved by the Swedish Research Ethics Board (etikprövningsnämnden, approved, Ref. No. 2019-03034). The sub-study with healthcare personnel interviews was exempt from review, since it focused on these interviewees’ work and work place in a way that did not address sensitive personal data (scientific secretary of this board; 2017-10-31).

3 Phenomenological philosophy is a more than a century old philosophical tradition (see e.g. the work of Edmund Husserl, Martin Heidegger, Simone de Beauvoir and Maurice Merleau-Ponty). Phenomenological philosophy has developed into several sub-fields such as that of phenomenology of medicine, feminist phenomenology and critical phenomenology. Further, combined insights from phenomenological work and contemporary neuroscience have informed the 4E Concept of Cognition (see Carney 2020).

4 Some of the interviewees seemed to be aware of their test results, but not all of them. However, as part of the routines at the clinic, patients were told the results by the occupational therapists performing and assessing the test. When discharged from the hospital, they were also to discuss the tests and their results with a doctor, according to the routine.
